# Validated Multi-Physical Finite Element Modelling of the Spot Welding Process of the Advanced High Strength Steel DP1200HD

**DOI:** 10.3390/ma14185411

**Published:** 2021-09-18

**Authors:** Konstantin Prabitz, Marlies Pichler, Thomas Antretter, Holger Schubert, Benjamin Hilpert, Martin Gruber, Robert Sierlinger, Werner Ecker

**Affiliations:** 1 Materials Center Leoben Forschung GmbH, Roseggerstraße 12, 8700 Leoben, Austria; marlies.pichler@mcl.at (M.P.); werner.ecker@mcl.at (W.E.); 2 Institute of Mechanics, Montanuniversitaet Leoben, Franz Josef-Straße 18, 8700 Leoben, Austria; thomas.antretter@unileoben.ac.at; 3 Mercedes-Benz AG, 71059 Sindelfingen, Germany; 4 Voestalpine Stahl GmbH, voestalpine-Straße 3, 4020 Linz, Austria

**Keywords:** resistance spot welding, finite element simulation, advanced high strength steels, phase transformation, zinc coated sheets

## Abstract

Resistance spot welding (RSW) is a common joining technique in the production of car bodies in white for example, because of its high degree of automation, its short process time, and its reliability. While different steel grades and even dissimilar metals can be joined with this method, the current paper focuses on similar joints of galvanized advanced high strength steel (AHSS), namely dual phase steel with a yield strength of 1200 MPa and high ductility (DP1200HD). This material offers potential for light-weight design. The current work presents a multi-physical finite element (FE) model of the RSW process which gives insights into the local loading and material state, and which forms the basis for future investigations of the local risk of liquid metal assisted cracking and the effect of different process parameters on this risk. The model covers the evolution of the electrical, thermal, mechanical, and metallurgical fields during the complete spot welding process. Phase transformations like base material to austenite and further to steel melt during heating and all relevant transformations while cooling are considered. The model was fully parametrized based on lab scale material testing, accompanying model-based parameter determination, and literature data, and was validated against a large variety of optically inspected burst opened spot welds and micrographs of the welds.

## 1. Introduction

Well fabricated spot welds are of great interest especially in the automotive industry, because typically up to 5000 spot welds are set per vehicle [1,2]. Therefore, an in-depth knowledge of the process is necessary to allow for designing welding systems and processes for reproducible and reliable welding joints while keeping the welding times short. The use of advanced high strength steels (AHSS) opens a big opportunity for light weight design of car bodies, which supports fulfilling the demands on resource economy and environmentally friendly transport. The high-ductility grades, which allow for better formability and increased safety, are especially gaining importance [3]. To increase the corrosion performance of these steels they are typically galvanized with zinc layers of about 7 µm thickness on both sides of the steel sheet. Resistance spot welding (RSW) processes are well established and yield high quality spot welds for AHSS grades with a typical strength level of 1000 MPa, and even higher for AHSS grades with lower ductility. However, if strength levels of 1200 MPa are approached, galvanized high ductility AHSS (AHSS-HD) grades like the dual phase steel with high ductility (DP1200HD, Manufacturer, city, country) investigated in the current study become susceptible to liquid metal embrittlement (LME) leading to liquid metal cracking (LMC). LME describes a drastic loss of ductility caused by intergranular [4,5,6,7,8] cracking for a specific material pairing and a specific thermal [3] and mechanical loading situation [9]. In order to understand, control and reduce the LMC risk of galvanized AHSS-HD the RSW process parameters have to be carefully chosen. Commonly, this is accomplished by means of extensive characterisation of an RSW joint and a thorough analysis of the quality of the resulting spot weld, while micrographs can then be correlated to process parameters such as welding current, welding time or electrode force. Naturally, the transient evolution of temperature, as well as metallurgical and mechanical fields are not accessible in these standard tests and hence the reason for local LMC remains unclear. The group of Yeong-Do Park [10,11] carried out sophisticated split RSW tests. While their research can be regarded as a major step towards elucidating the internal mechanisms, the internal state variable still cannot be determined accurately since the testing set-up influences the local conditions. So far, the only viable approach that allows monitoring of these transient fields is a numerical process simulation by means of the finite element (FE) method. The literature reports on a number of FE models of RSW processes [12,13,14,15,16]. In most of those publications only the electrical-thermal-mechanical coupling is considered, whereas the metallurgical and mechanical effects of phase transformations are not covered [17] or only dealt with by applying some simplifications [18,19]. Since the LMC forms during the heating phase of the process while the centre of the spot weld has already melted and consequently the adjacent area has transformed from the base microstructure to austenite [10], the effects of the phase transformation must not be neglected. It is important to point out that the phase transformation not only changes the metallurgical field but also affects the thermal and mechanical fields. The work of [20] considers the temperature path in a reasonable way, but phase dependent material properties were not fully investigated and were therefore only partially implemented. Other publications [14,21] base their model merely on material properties gathered from the literature. In the literature, modelling of residual stresses can be found for, e.g., butt welded plates [22], thermal-vibratory stress relief processes [23], laser-magnetic welding [24], as well as steel tubes [25].

In the present work a multi-physical RSW model is presented that couples electrical, thermal, mechanical, and metallurgical phenomena and is capable of calculating the transient fields of e.g. current densities, temperature, stresses, elastic and plastic strains, and the phase state. With the model, the residual stress state in the final spot weld can also be calculated. The rigorous approach found in this work by modelling and validating the RSW process in great detail exceeds current publications. The model is built in a bottom-up way based on material parameters determined in laboratory tests. This involves many strain-rate dependent flow curves as well as the thermal properties for all relevant material phases and a wide temperature range. These material data serve as input for the numerical model, whose results are validated against a large number of weld tests via optical macro-inspection of burst opened spot welds, and via light microscopy of selected micrographs. In the validation step the parameters for the electrical contact model, which are hard to determine in a direct way, are adapted to improve the predictive quality of the model. Eventually, the model is used to investigate the influence of varying welding parameters on the quality of the spot welds. This leads to a validated model which accounts for phase transformations during the whole process including the heating and cooling sequence. Furthermore, the model can be used to optimize the welding process with the goal of reducing the risk of LMC as characterized by an appropriate damage criterion such as the one reported in [26].

## 2. Methodology

### 2.1. Spot Welding Process

**Figure 1:**
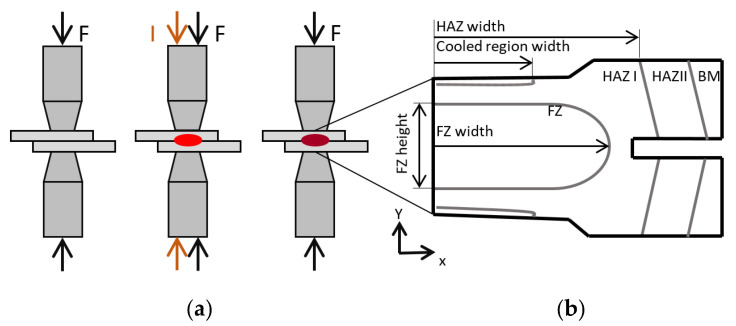
(**a**) RSW process steps: 1st clamping of electrodes and applying the welding force, 2nd initiating the welding current and 3rd hold time, (**b**) the different zones of the weld and LME locations [15].

The RSW process can be subdivided into three subsequent process steps. In the first step the two concentrically aligned electrodes—in the current case made of copper alloy (CuCrZr) [18]—move perpendicular to the sheets [27]. After contacting the sheets, a defined electrode force *F* is applied, and the sheets are pressed together, as shown in Figure 1a. In the next step a welding current *I* is turned on, which leads to joule heating. Due to the high interface resistivity, compared to the bulk electric resistivity, the joule heating is most pronounced at the interface between the sheets, which leads to a molten zone called the fusion zone (FZ). A smaller amount of heat is generated at the interfaces between sheets and electrodes as well as in the sheets themselves [28]. Electrode force and elevated temperatures lead to a subsequent change of contact conditions and to a change of heat generation. This triggers different metallurgical phase transformations, which lead to an evolution of material properties represented by their flow curves and mass densities. Furthermore, latent heat is released. The welded sheets remain clamped between the electrodes during a defined holding time in order to keep the cooling rates high before they are finally released.

The size of a well-executed FZ providing a proper connection is defined by a minimum diameter $d=4\times \sqrt{t}$ [29], where t accounts for the sheet thickness, which is in the current case 1.6 mm. The maximum current and hence the maximum diameter is limited by the expulsion of molten steel due to overheating. The different characteristic zones of the weld are the FZ, upper critical heat affected zone (HAZ I), sub-critical heat affected zone (HAZ II), and the unaffected base material (BM) as shown in Figure 1b [30,31,32,33]. Additional important geometrical quantities used for validation are marked in this figure. The zone named “cooled region” is the zone near the water-cooled electrodes which does not undergo a phase transformation.

### 2.2. Experimental

The investigated DP1200HD (Voestalpine Stahl GmbH, Linz, Austria) steel shows a chemical composition given in Table 1 measured by means of optical emission spectrometry. The microstructure consists of about 50% ferrite, 40% martensite, and 10% retained austenite.

The temperature dependent thermo-physical data were determined at the Oesterreichisches Giesserei Institut, Leoben, Austria. Density *ρ* was measured with a Sartorius ED224S (Sartorius AG, Göttingen, Germany)while the thermal expansion *α_CTE_* was investigated with a Netzsch DIL 402 CD (NETZSCH-Gerätebau GmbH, Selb, Germany). From the measurement of the specific heat c*_p_* with a Netzsch DSC 404 (NETZSCH-Gerätebau GmbH, Selb, Germany), the specific latent heat *L_S_* was calculated. For determining the phase transformation kinetics, dilatation experiments with the Netzsch DIL 402 CD were carried out. Plastic material data for the base material and a purely austenitic microstructure were obtained via hot tensile testing with a Gleeble® 3800 (Dynamic Systems Inc., Poestenkill, NY, USA) at the Graz University of Technology, Graz, Austria. In addition, the Koistinen–Marburger coefficient governing the martensite formation kinetics was also determined from non-isothermal tests on the same facility. 

For an extensive model validation instrumented RSW, tests were conducted on an X-calliper and a Nimak pedestal welder (NIMAK GmbH, Wissen, Graz) with a Matuschek controller (Matuschek Meßtechnik GmbH, Alsdorf, Germany) and a C-calliper, respectively, for similar 1-, 2-, and 3-sheet stack-ups of DP1200HD sheets. Table 2 reports the welding schedule for all test welds provided in the paper. The duration of the single pulse direct current, i.e., the welding time *t_w_* is set to be between 190 ms and 760 ms and the electrode force *F* between 3.5 MPa and 5.3 MPa. The holding time *t_h_* varies from 100 ms to 900 ms. With every parameter set, three identical welding tests were produced. Note that the appropriate welding current *I* was determined for the 2-sheet stack-up and then also applied to 1- and 3-sheet stack-ups for comparison reasons. All welding specimens were optically examined, and micrographs were taken for selected samples. The micrographs were Nital etched. The characteristic geometrical and microstructural features documented in Figure 1 were digitized based on optical light microscopy investigations to serve as validation of the numerical simulations. Finally, XRD (X-ray diffraction) measurements were conducted in the spot weld centre. The irradiated spot width of the XRD was 1 mm square while the stress measurement was carried out perpendicular to the sheet surface. Vickers hardness distribution with the microhardness HV1 was monitored according to EN ISO 6507-1 [34].

### 2.3. Modelling

#### 2.3.1. General Aspects

**Figure 2:**
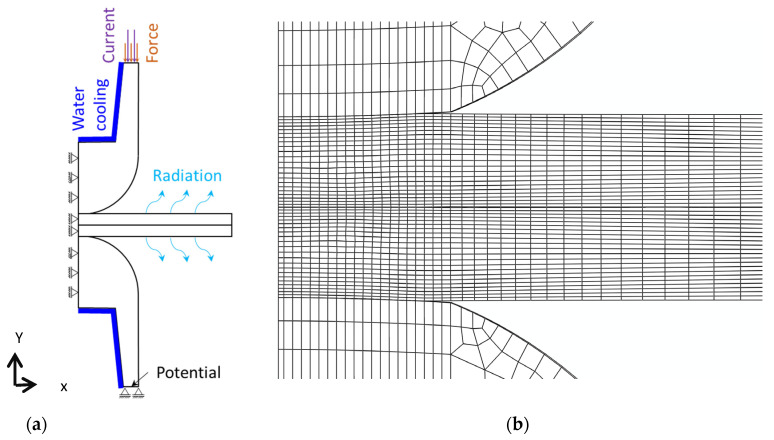
(**a**) Applied boundary conditions and loadings, (**b**) mesh of a 2-sheet model.

Figure 2a depicts the schematic geometry of a representative model showing a 2-sheet stack-up. In their initial position the two electrodes contact the steel sheets in the model. The sheet thickness is 1.6 mm. The investigated electrodes of the type F1-16-20-6 are further specified in ISO 5821 [35]. The cavity inside the electrodes allows for internal water cooling, while the sheet width is 20 mm.

For the finite element (FE) modelling the commercial software package ABAQUS/Standard version 2019 [36] was applied. Since the software does not support calculation of 2D axi-symmetric coupled electrical-thermal-mechanical problems and to keep the numerical effort low a 3D wedge model with an aperture angle of 3° and axi-symmetric boundary conditions was created. Hexahedral elements Q3D8 and wedge elements Q3D6, both with linear shape functions were used and the minimum element size in the model was set to 50 microns. The wedge elements were only needed for discretizing the element row at the axis of the electrode of the model. The mesh is shown in Figure 2b and it is worth mentioning that for convergence reasons a matching mesh between electrodes and sheets turned out to be beneficial. The time evolution of the RSW process was structured in the FE model in four simulation steps, i.e., the three process steps shown in Figure 1 and an unloading step where the contact between electrodes and sheets is released. The loading conditions and boundary conditions are depicted in Figure 2a. At the upper electrode, force and current were applied with a time evolution as described in Figure 1 and values according to the welding schedules given in Table 2. The lower electrode was fixed with respect to its displacement in the y-direction and the electrical potential was set to zero Volt. The water cooling of the electrodes was modelled by means of a convective heat transfer condition. A heat transfer coefficient of 7.5 mW/(mm^2^K) was taken from [37], assuming turbulent flow conditions and a mass flow of 6 litres per minute. The cooling water temperature was 25 °C. The radiation at the sheet surfaces was taken into account with an emissivity of 0.2 which was reported in the literature for galvanized steel sheets [38]. Due to the very short process time, natural convection at the sheet surfaces as well as at the electrodes has very little effect and thus, was not considered in the model. Coulomb friction with a friction coefficient of 0.1 was used within the surface-to-surface contact algorithm between the sheets and between electrode and sheet. Electrical contact conductivity and thermal contact conductivity between the sheets and between sheets and electrodes for electrogalvanized sheets were taken from the literature [26]. The electrical contact conductivity was subsequently readjusted by means of inverse parameter optimization. The contact properties are summarized in Table 3. 

#### 2.3.2. Material Model

The multi-physical material model couples the electrical-, thermal-, metallurgical- and mechanical problems. The electrical and thermal problem was solved with the built-in model capabilities of the commercial software package ABAQUS/Standard (version 2018, Dassault Systèmes, Vélizy-Villacoublay, France) whereas the metallurgical and mechanical material model equations were implemented by means of user-defined subroutines.

**Figure 3:**
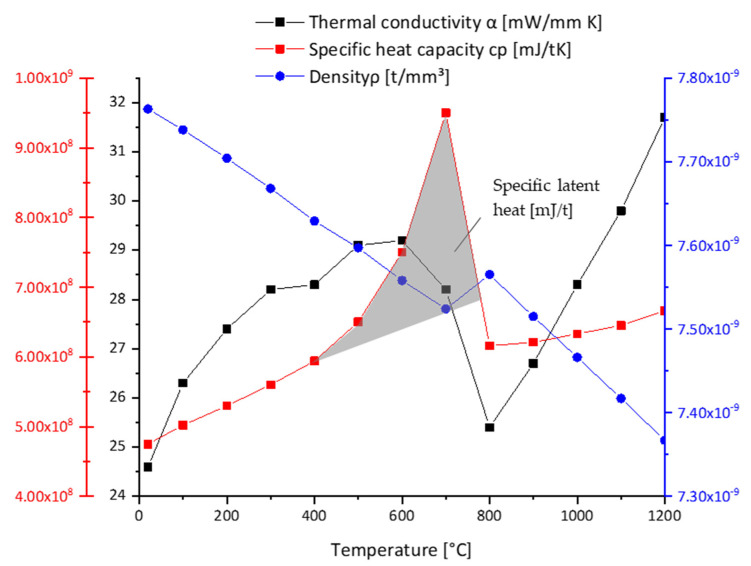
Specific heat (*c_p_*), latent heat, density (*ρ*) and thermal conductivity (*α*) over temperature.

The measured temperature dependent thermal conductivity *λ*, the specific heat capacity *c_p_*, and the density *ρ* are documented in Figure 3 and a list of the implemented phase dependent values is provided in Table 4. 

For the sake of simplicity, the same material properties were applied for the base material and the martensitic phase and both are in this respect referred to as low temperature phase. The specific latent heat for the solid-to-solid phase transformation *L_S_* was extracted from the calorimetric (DSC) measurement by calculating the area under the *c_p_*–*T* curve relative to the linearly extrapolated *c_p_*–*T* curve of the parent phase (see shaded area in Figure 3). The so-calculated specific latent heat *L_S_* was 9.1 × 10^10^ mJ/t. The specific latent heat for the solid to liquid phase transformation *L_L_* was taken based on the literature as 20.5 × 10^10^ mJ/t [39]. It was assumed that for all phase transformations the specific latent heat fraction scales linearly with the transformed phase fraction. The electrical conductivity *σ*_e_ of the base material was calculated from the thermal conductivity *λ* using the Wiedemann–Franz law [27] given in Equation (1), where *T* is the temperature and *L* is the Lorentz number = 0.0244 mWmΩ/K^2^. This data was then used as the initial guess in a first calculation run and subsequently adjusted by means of inverse parameter optimization. The temperature dependent electrical material conductivity $\sigma_{e}$ is summarized in Table 5.

Since the difference between the electrical conductivity around the phase transformation temperature of the low temperature phase and austenite turned out to be similar a dependency on the phase state was omitted.

(1) $$\sigma_{e}=\frac{\lambda }{L\;T}$$

As described, joule heating causes the formation of a fusion zone between the sheets. The joule heating follows Equation (2), where *Q_W_* stands for the created thermal energy, *I* for the current, *R* for the electrical material resistance, and *t* for time [2]. For contact heating the electrical contact resistance *R_c_*, which is significantly higher than the electrical contact resistance *R*, has to be considered in the calculation instead of *R*.

(2) $$Q_{W}=I^{2}\;R\;t$$

A special focus of this work was set on the phase transformation model and its thermal and mechanical consequences based on strain rate and phase dependent material data, since this has not been treated in the literature in a similar rigorous form to that presented here. The phase transformation model was implemented by means of a set of user-defined Fortran 77 subroutines that combine four main aspects: (1) the phase transformation kinetics, (2) the latent heat generation, (3) the metallurgical strain caused by phase transformation, and (4) the formation of transformation induced plasticity. Furthermore, the thermal and mechanical material properties are modelled to be dependent on the phase fractions. The considered phases are the low temperature phase representing the base material and the martensitic state, the austenitic phase, and the liquid phase. The material properties of phase mixtures are calculated by means of a linear rule of mixture.

**Figure 4:**
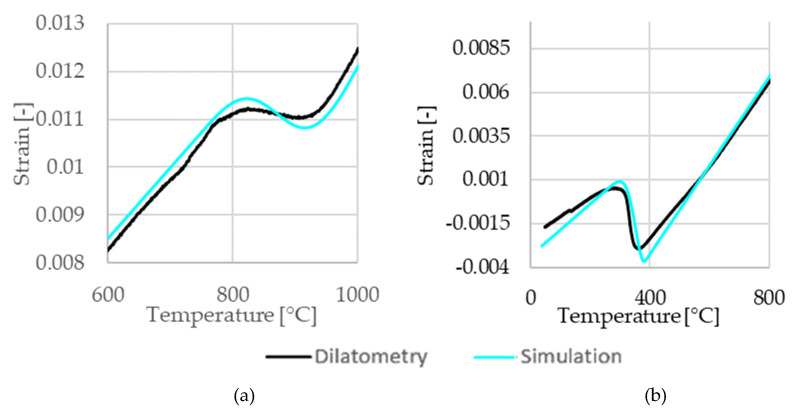
Comparison of the dilatometer measurements with the model predictions (Sim) for (**a**) heating at 400 °C/s and (**b**) cooling at 100 °C/s.

The phase transformation kinetics model for the solid-to-solid phase transformations is calibrated based on dilatometric tests with temperature rates of 3 °C/S, 100 °C/s and 400 °C/s. Figure 4 shows the heating and cooling sequence of the experiment with a heating and cooling rate of 100 °C/s. Black lines indicate measured data and cyan lines indicate the respective simulation results. The phase changes of base material to austenite, austenite to liquid, and liquid to austenite are treated as diffusive phase transformations. The Scheil [29] approach was used to convert the data obtained from isothermal TTT diagrams to continuous cooling conditions as prevalent in the actual welding process. Additionally, the Johnson–Mehl–Avrami–Kolomogorov (JMAK) [40] kinetics given in Equation (3) was used, where *f_x_* is the phase fraction of the newly created phase, *t* is the time of isothermal holding, *k* accounts for an overall rate constant, and *n* is the Avrami coefficient.

(3) $$f_{X}\left(t\right)=1-\exp\left(-kt^{n}\right)$$

The parameters applied for the JMAK model for solid-to-solid phase transformation from base material to austenite were *k* = 6.65 × 10^3^ and *n* = 2 at a rate of 1000 °C/s, for austenite–liquid transformation *k* = 5 × 10^3^ and *n* = 2 and for liquid–solid transformation *k* = 6.65 and *n* = 2. The kinetics parameters of the latter two phase transformations were determined based on ThermoCalc 2017 (Thermo-Calc Software AB, Solna, Sweden) calculations.

The Koistinen–Marburger kinetics [41], shown in Equation (4), was chosen to describe the solid state transformation from austenite to martensite. *f_M_* is the volume fraction of martensite, *M_S_* is the martensite start temperature, *T* describes the current temperature, *f_γ_* is the volume fraction of austenite, and *a* is the Koistinen–Marburger parameter [41]. A Koistinen–Marburger parameter of 0.011 led to a good match between the experimental results and the model. Due to rapid cooling no other phases besides martensite are formed.

(4) $$f_{M}\left(T\right)=f_{\gamma }(1-\exp\left(a\left(M_{s}-T\right)\right)$$

The mechanical problem is formulated in the small strain regime. Hence, the total strain tensor $\varepsilon^{tot}$ can be decomposed into different strain contributions as seen in Equation (5). In the present case these strain contributions are elastic strain $\varepsilon^{el}$, thermal strain $\varepsilon^{th}$, metallurgical strain $\varepsilon^{me}$, plastic strain $\varepsilon^{cp}$ (due to classical plasticity), and transformation induced plasticity strain $\varepsilon^{tp}$.

(5) $$\varepsilon^{tot}=\varepsilon^{el}+\varepsilon^{th}+\varepsilon^{me}+\varepsilon^{cp}+\varepsilon^{tp}$$

The elastic material properties were defined for both the low temperature phase and the austenitic phase in a temperature dependent form. The Young’s moduli and the Poissons’s ratios were taken from [42] and [25], respectively. For the liquid phase a quasi-solid elastic body with a Poisson’s ratio of 0.48 and a Young’s modulus of 1 GPa was assumed. The elastic properties are summarized in Table 6. 

**Figure 5:**
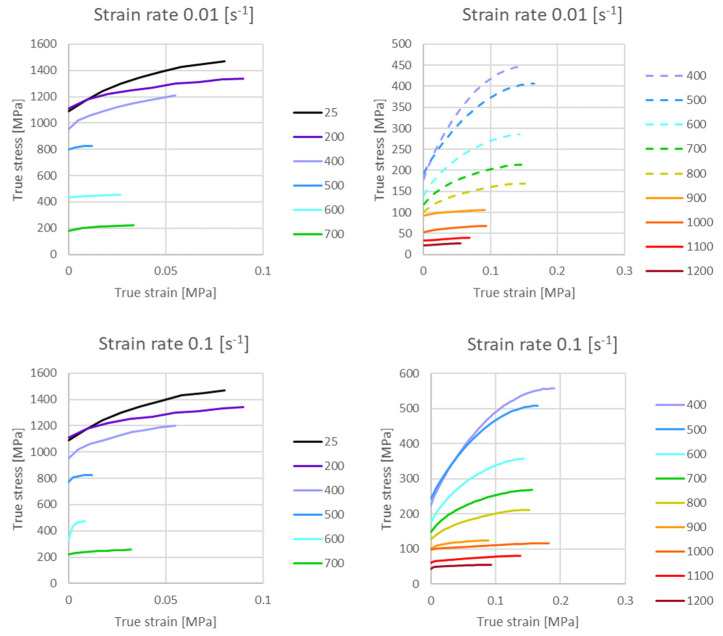

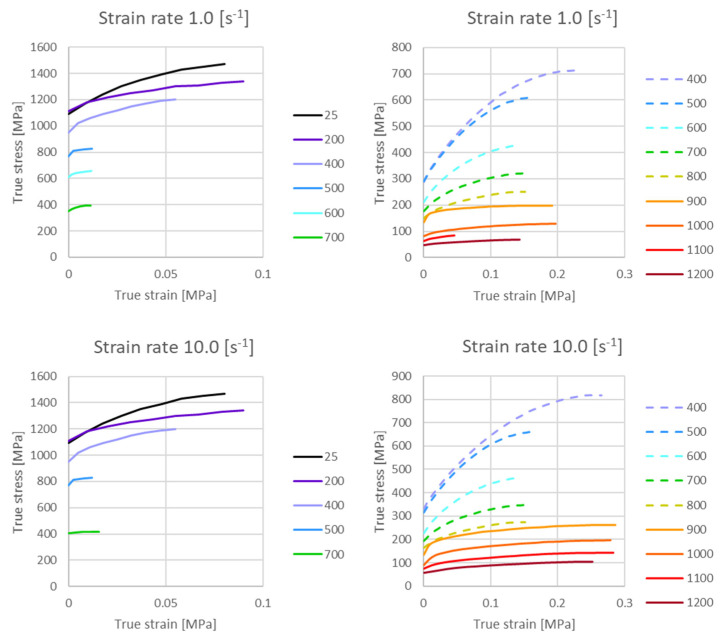
Plastic flow curves for strain rates of 0.01 s^−1^, 0.1 s^−1^, 1.0 s^−1^, 10 s^−1^ for the base material (**left**) and for austenite (**right**), dashed lines show extrapolations (**right**).

The mean coefficient of thermal expansion *α_CTE_* was measured to be 1.478 × 10^5^ K^−1^ between 20 °C and 500 °C for the base material and 2.52 × 10^5^ K^−1^ for temperatures ranging from 900 °C to 1200 °C for austenite. For the liquid steel melt which appears above about 1500 °C the thermal expansion coefficient was taken from the literature as 4.0 × 10^5^ K^−1^ [43]. The metallurgical strain between the low temperature phase and the austenitic phase accounts for the transformation dilatational strain, however, corrected for the thermal expansion in the transformation window [44]. The plastic yield stress and the isotropic hardening evolution were defined by means of tabular data depending on temperature, strain rate and phase type. The plastic flow curves were measured for the low temperature phase (base material) and austenite for temperatures up to 700 °C and 1200 °C, respectively, and for four strain rates between 0.01 s^−1^ to 10 s^−1^. The set of implemented flow curves is shown in Figure 5. The dashed lines indicate extrapolated data for the metastable austenite following the procedure proposed in [25].

The rate of the accumulated strain is abbreviated as strain rate. The transformation induced plasticity strain tensor $\varepsilon^{tp}$ is in the current case only relevant for the austenite to martensite transformation. It was calculated according to Equation (6), with S as the stress deviator, f′(ξ) as the saturation function with a maximum of 1, $\dot{\xi }$ describes the time derivative of the volume fraction of the product phase and Δt denotes the time increment [44]. The Greenwood-Johnson parameter was determined experimentally from dilatometry under load to be $K_{M}=5.18$ × 10^5^ MPa^−1^.

(6) $${\dot{\varepsilon }}^{tp}=\frac{3}{2}K_{M}Sf\prime \left(\xi \right)\dot{\xi }$$

The material data for the copper–chrome–zircon electrodes were taken from the literature [45,46].

The full set of mainly experimentally determined model parameters and the consideration of the evolution of the metallurgical fields and their related quantities governed by phase transformation kinetics models allow a realistic model description of the RSW process and the evolution of the local quantity fields.

## 3. Results

Basically, changing the welding parameter led to the same trends in the model as in the experimental. Higher current plus prolonged welding time leads to a larger FZ and HAZ due to the increased heat input. Elevated electrode forces help enhance the contact between the sheets and electrodes, while resulting in a better containment of the liquid FZ. It allows higher welding currents, but on the downside a deeper electrode sink in occurs. The holding time after welding is essential for cooling the spot weld but has the smallest impact on the weld formation.

**Figure 6:**
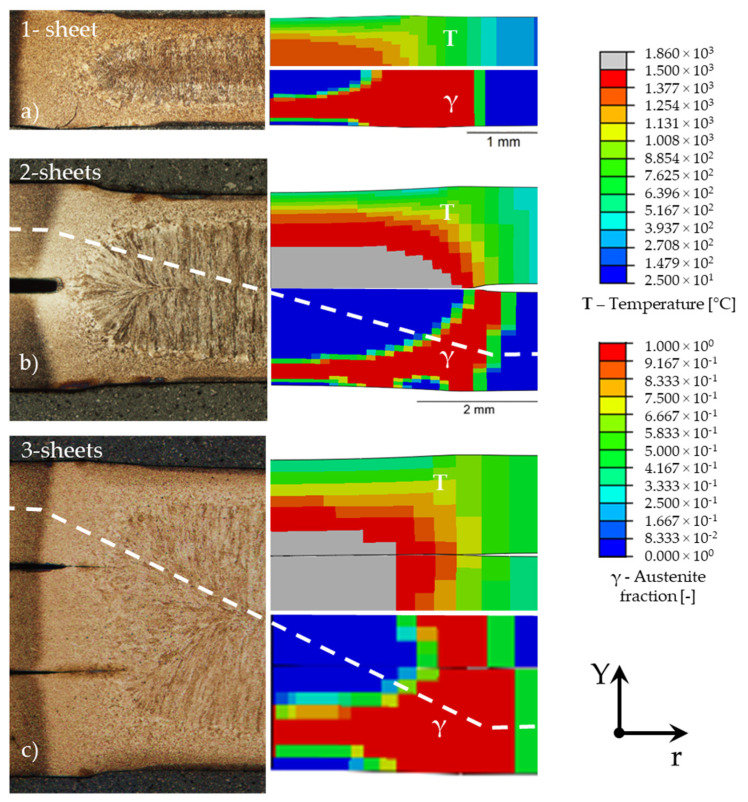
Micrographs (left column) and simulation results (right column) of (**a**) 1-, (**b**) 2- and (**c**) 3-sheet stack ups for *I* = 6.6 kA, *F* = 4.5 kN, welding time *t_w_* = 380 ms, holding time *t_h_* = 300 ms (configuration 23 in Table 2).

Figure 6 shows the experimental and numerical results for different sheet stack-ups with the same welding conditions (welding current *I* = 6.6 kA, electrode force *F* = 4.5 kN, welding time *t_w_* = 380 ms, holding time *t_h_* = 300 ms). A 1-sheet stack up as seen in Figure 6a was used to fine-tune the electrical contact conductivity between the electrode and sheet and the electrical resistance in the steel by means of inverse modelling. The contact conditions between the sheets were calibrated with a 2-sheet stack-up model depicted in Figure 6b. The model for 3-sheet stack-ups as in Figure 6c was used to verify the previous findings. It is important to point out that the above-described model calibration procedure was carried out for a X-calliper, whereas the results provided in Figure 6 were obtained with equal welding parameters but on a different RSW rig with a C-calliper. That way the validity of the model was put to test. For each sheet stack up an assembly of experimental and numerical results is depicted. The left-hand side shows a micrograph of a cross section through the centre point of the spot weld enabling the investigation of its heterogeneous microstructure. The upper right figure displays the temperature distribution at the end of the welding time *t_w_*, when the highest temperatures occur. The lower right contour plot shows the corresponding austenite fraction at *t_w_*. As expected, temperatures are highest in the centre of the spot weld, where the FZ is formed due to localized melting of the steel. In the micrograph this zone is indicated by bright etching and coarse grains, elongated along the direction of the highest temperature gradient. In the plot showing the austenite fraction the FZ corresponds to the blue area at the centre of the sheet stack-ups. Next to the FZ the upper critical HAZ I is located where austenite forms but the material does not melt. In this zone martensite forms during subsequent rapid conductive cooling by the surrounding steel sheet areas. This zone is indicated by bright etching and a fine microstructure in the micrographs and shows an austenite fraction of 1 in the simulation. The sub-critical heat affected zone (HAZ II) in the micrograph cannot be differentiated from the HAZ I. In the simulation, however, it is visible as the green coloured zone with an austenite fraction between 0 and 1 near the base material. At the contact surface to the electrodes a dark etching area is visible in all three micrographs. This distinct area is also found in austenite fraction plots. The material in this zone is not heat affected due to the cooling from the electrodes. Since the three investigated sheet stack-ups were welded with identical welding parameters the influence of the sheet stack-up size can be analysed. The overall maximum temperature in the centre of the FZ increases with the number of welded sheets, but the surface temperature decreases and the heat unaffected zone near the surface is largest with the 3-sheet stack-up spot weld. For the quality of the developed model, it is important to note that for all three stack-ups a very good agreement for the lateral extension of the different zones across the weld was achieved. 

**Figure 7:**
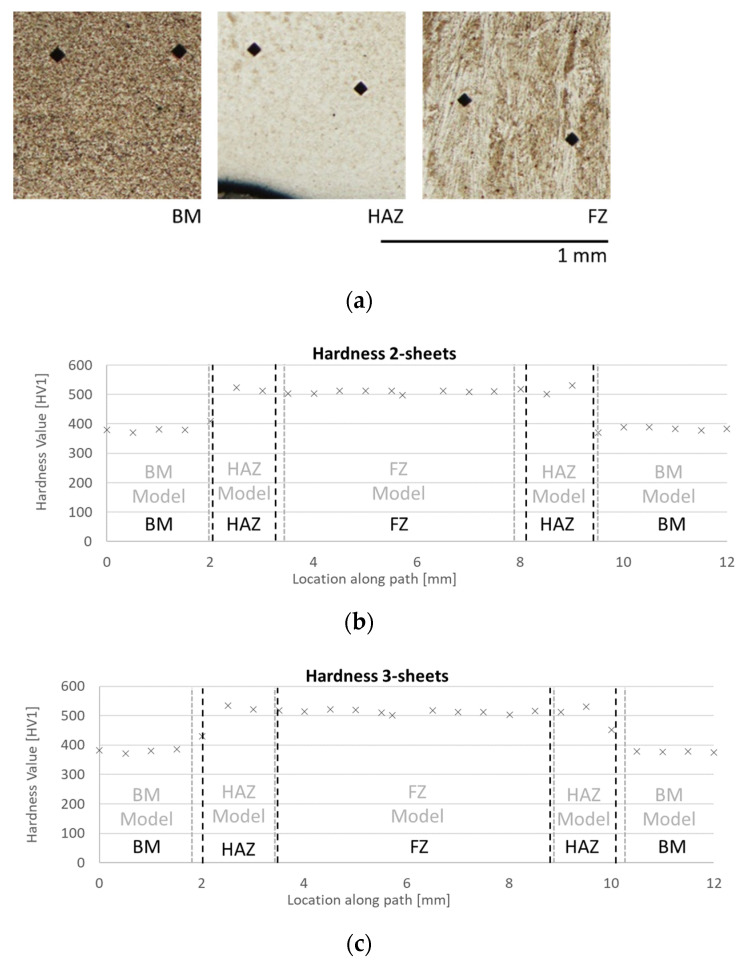
(**a**) Microhardness measurements at different spot weld areas. The microhardness values (indicated by × markers) for the (**b**) 2-sheet stack up and (**c**) 3-sheet stack up in comparison to the respective location of all different metallurgical zones as found by image analysis of the micrographs (indicated in black) and to the zone boundaries predicted by the model (indicated in grey).

To verify these findings, microhardness profiles of the 2- and 3-sheet stack ups were recorded along the path indicated in Figure 6b,c. Figure 7 shows the difference between the base material, with hardness values of slightly below HV 400, and the HAZ and the FZ with hardness values well above HV 500. Dual phase steels such as the investigated DP1200 here are relatively resistant against HAZ softening [47]. The present results support these findings. At the given lateral resolution of about 0.5 mm only in the 2-sheet stack up, just the indent at 8.7 mm shows a small softening by about 5%. The FZ can clearly be differentiated from the HAZ by its solidification structure with elongated grains occurring only in the FZ. A comparison of the different zone boundaries as identified from the micrographs as well as the microhardness profiles with the model predictions is given in Figure 7b,c. Zones of different grain sizes as well as grain morphologies were identified by automatically adapting the image threshold in ImageJ (Wayne Rasband, Bethesda, MD, USA). Their boundaries perfectly match the zone boundaries determined from the microhardness profiles.

The RSW reacts sensitively to different inputs such as welding current or electrode force. Since the presented RSW model will in the future be applied for process design purposes the model has to be robust in its predictive quality with respect to the local physical quantities. Hence, further validation of the model is necessary. To this end simulations for 2- and 3-sheet stack-ups with the complete list of welding schedules shown in Table 2 were carried out and assessment quantities for the experimental tests were digitized. In a first validation set the model was validated against a geometrical characterization of 37 burst opened spot welds that had been joined using an X-calliper, and in a second set the model was validated against 23 micrographs of spot welds created with both kinds of callipers. The specified assessment quantities are also key quantities for a proper spot weld, i.e., the fusion zone width, the fusion zone height, the width of the cooled region under the electrodes, and the width of the HAZ. These assessment quantities are all illustrated schematically in Figure 1b. The scatter plots in Figure 7 show a comparison of the assessment quantities from experiment and simulation. In Figure 7a the FZ width and the penetration depth of the electrodes are compared in the first validation set. The FZ width shows a very good agreement for the 2-sheet stack-ups and a reasonably good agreement for the 3-sheet stack-ups. The standard deviation between measured and calculated FZ widths is 2% and the maximum relative deviation is 15%. The overall relative deviation of the sum of all assessment points is only 2%. The modelled electrode penetration depths agree well in their tendency but are underestimated by a factor of 2. As explanation the squeeze out of liquid steel was identified which is rather difficult to implement in this kind of model. Nevertheless, it is irrelevant for calculating the phase distribution.

**Figure 8:**
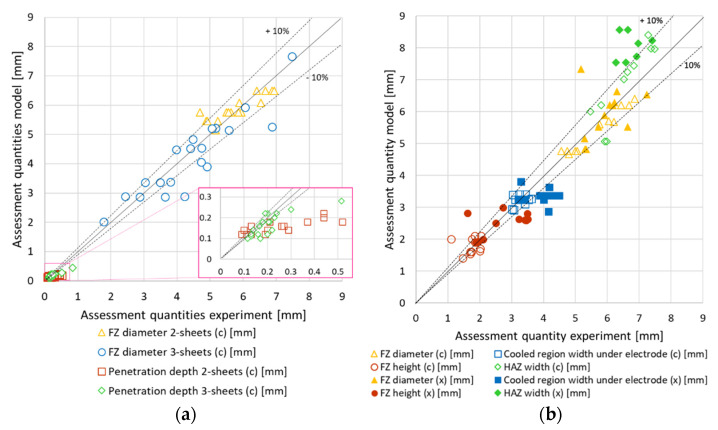
Comparison of the model and experiments for (**a**) burst opened and (**b**) micrographs while (c) marks welds done with the C-calliper and (x) marks welds with the X-calliper.

In the second validation set, the micrographs were analysed for 23 spot welds. This allows comparison besides the FZ diameter further assessment quantities. For the comparison in Figure 8b, 2- and 3-sheet stack-ups welded on two different welding machines and with the X- and C-calliper were examined. The investigated features of the spot welds produced with the two different callipers compare well. The results of the welds with the C-calliper match a little better to the model than those with the X-calliper. The reason for this is that the C-calliper shows a better alignment while the X-calliper tends to tilt the electrodes slightly. For the sake of simplicity, perfect alignment with no tilt of the electrodes with respect to each other was assumed. Looking at the results in more detail, one sees a good match of the FZ diameter and the FZ height. The comparison of the width of the cooled region underneath the electrodes shows reasonable agreement, with a slightly higher spread of the experimental data than of the simulation results. The size of the heat affected zone tends to be overestimated by the simulations by about 15%. The reason for this might be the rather coarse mesh size of about 0.2 mm near the boundary of the HAZ unable to resolve the sharp gradients of the field variables at that point. For the second validation set, the mean deviation between the model and the experiment was found to be 2% for all assessment points.

**Figure 9:**
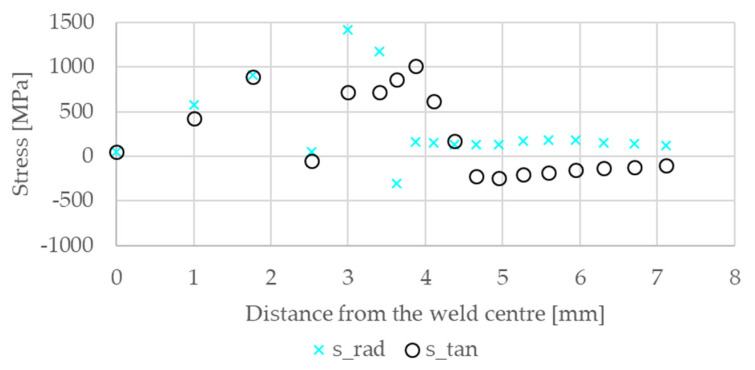
The simulated residual stresses in the radial and tangential direction are shown at the spot weld surface.

Measured residual stress distributions for the radial stress *σ_r_* and the tangential stress *σ_t_* were found in the literature of Iyota et al. [19] for a HT980 steel grade. In that paper, the used sheet thickness of 1.6 is the same but the welding parameters differ with respect to the present investigation. In ref. [19] the welding current was 6.0 kA, the electrode force 3.5 kN and the welding time 280 ms, while in the present case the welding current was 6.6 kA, the electrode force 4.5 kN, and the welding time 380 ms (configuration 23 in Table 2). Hence, the comparison can only be qualitative. However, the measured radial stress *σ_r_* and tangential stress *σ_t_* along a radial path at the sheet surface compare in their trends well to the results of the current model. Iyota et al. measured local positive minima at a distance of about 2.1 mm from the centre while local positive maxima were shown at approximately 1 mm and 3 mm. The slight difference in these positions occurs due to the different electrodes used and the welding conditions. Due to the higher yield strength of the DP1200HD residual stresses are higher than found in that paper, see Figure 9. 

**Figure 10:**
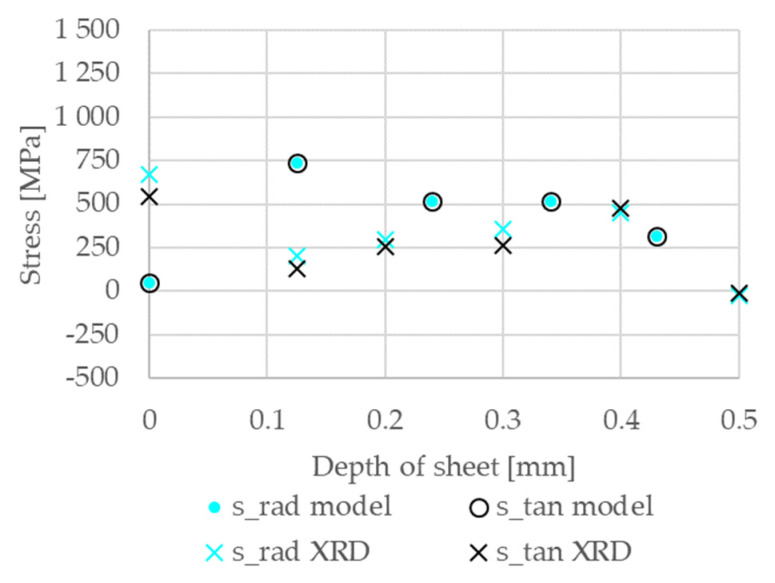
Simulation results for the conditions after rapid cooling compared to residual stresses in radial (s_rad XRD) and tangential direction (s_tan XRD) of XRD measurements in the sheet depth for the centre of the weld. Note that the calculated radial s_rad model and tangential s_tan model stress distribution overlap.

For further validation residual stress measurements were done at the centre of the weld of configuration 23, see Figure 10. The radial ($\sigma_{r}$) and tangential ($\sigma_{t}$) stress results of the simulation and measurement are in reasonable agreement. Around the zero-position, the surface effects are the reason for the differences.

Due to the good overall correlation the model can be rated as validated. The model might be further applied to increase the understanding of the evolution of local quantities during RSW and it can be used for RSW process design. This is especially interesting for minimizing effects such as LME by means of improved process parameters and electrode geometries. For further improvement of the simulation results it would be necessary to determine the electrical properties of the steel sheets over a large temperature range for this steel grade.

## 4. Conclusions

A large amount of material data describing the electrical, thermal, metallurgical, and mechanical properties of the advanced high strength dual phase steel DP1200HD necessary for simulating RSW processes was collected in lab scale material tests. These data served as input for a multi-physical FE-model. At the same time, a large number of resistance spot welds were produced for a broad spectrum of different welding conditions, welding process parameters, sheet stack-up size, welding guns, and welding machines. The spot welds were measured and micrographically analysed. The FE model was validated by matching the temperature and phase distributions to these micrographs, by comparing the numerical results to especially defined assessment quantities of 60 different spot welds. The numerical model accounts for complex phase transformation phenomena including the mechanical consequences due to the phase state dependent material properties, volume jumps during transformation, and transformation induced plasticity. The model thus offers the opportunity to analyse the evolution of local thermal and mechanical quantities and enables knowledge-based process design. It is a step towards designing resistance spot welding processes with optimum fusion zone formation which will be important for the reduction of liquid metal cracking around spot welds.

## Figures and Tables

**Table 1:** Chemical composition of the DP1200HD steel and main alloying elements.

C [%]	Si [%]	Mn [%]	Cr [%]	Cu [%]	Nb [%]	Fe
0.21	1.46	2.53	0.03	0.016	0.002	balanced

**Table 2:** Spot weld testing schedule applied for 1-, 2- and 3-sheet stack ups, respectively.

C-calliper	Configuration	1	2	3	4	5	6	7	8	9	10	11	12
	Electrode force [kN]	4.5	4.5	4.5	3.5	3.5	3.5	5.3	5.3	5.3	4.5	4.5	4.5
	Welding-time [ms]	380	380	380	380	380	380	380	380	380	190	190	190
	Welding current [kA]	5.2	6.9	6.1	5.1	6.2	5.7	5.5	7	6.3	6.6	7.3	7
	Holding-time [ms]	300	300	300	300	300	300	300	300	300	300	300	300
C-calliper	Configuration	13	14	15	16	17	18	19	20	21			
	Electrode force [kN]	4.5	4.5	4.5	4.5	4.5	4.5	4.5	4.5	4.5			
	Welding-time [ms]	760	760	760	380	380	380	380	380	380			
	Welding current [kA]	5.2	6.9	6.1	5.4	6.9	6.2	5.4	6.9	6.2			
	Holding-time [ms]	300	300	300	100	100	100	900	900	900			
X-calliper	Configuration	22	23	24	25	26	27	28	29	30	31	32	33
	Electrode force [kN]	4.5	4.5	3.5	3.5	5.3	5.3	4.5	4.5	3.5	3.5	5.3	5.3
	Welding-time [ms]	380	380	380	380	380	380	760	760	760	760	760	760
	Welding current [kA]	7.3	6.6	6.6	6.1	7.3	6.8	7.5	6.7	7.0	6.1	7.6	6.8
	Holding-time [ms]	300	300	300	300	300	300	300	300	300	300	300	300

**Table 3:** Contact properties between electrode/sheet and sheet/sheet.

	Electrode to Sheet Contact		Sheet to Sheet Contact	
Temperature	Electrical Conductance	Thermal Conductance	Electrical Conductance	Thermal Conductance
°C	1/(mOhm mm^2^)	mW/(mm^2^ K)	1/(mOhm mm^2^)	mW/(mm^2^ K)
25	2	100	0.08	250
250		500		1000
350			0.08	
400	3			
420	36			
500	200			
530	220			
650			0.09	
1250			0.1	
1500	220		0.2	
1800		4000		4500

**Table 4:** Temperature dependent thermal conductivity λ, specific heat capacity c_p_, and density ρ.

Temperature	Conductivity	Specific Heat	Density
°C	mW/mm K	mJ/t K	t/mm^3^
**Low Temperature Phase**			
20	24.6	4.75 × 10^8^	7.76 × 10^−9^
100	26.3	5.02 × 10^8^	7.74 x 10^−9^
200	27.4	5.30 × 10^8^	7.70 × 10^−9^
300	28.2	5.60 × 10^8^	7.67 × 10^−9^
400	28.3	5.94 × 10^8^	7.63 × 10^−9^
500	29.1	6.50 × 10^8^	7.60 × 10^−9^
600	29.2	7.20 × 10^8^	7.56 × 10^−9^
700	29.5	8.00 × 10^8^	7.52 × 10^−9^
800	29.9	9.00 × 10^8^	
900	30.4	1.00 × 10^8^	7.45 × 10^−9^
**Austenite**			
400		5.82 × 10^8^	
500		5.89 × 10^8^	
600	22.0	5.95 × 10^8^	7.66 × 10^−9^
700		6.03 × 10^8^	
800		6.16 × 10^8^	7.56 × 10^−9^
900		6.21 × 10^8^	7.52 × 10^−9^
1000	28.3	6.33 × 10^8^	7.47 × 10^−9^
1100	29.8	6.45 × 10^8^	7.42 × 10^−9^
1200	31.7	6.66 × 10^8^	7.37 × 10^−9^
1300	33.3	6.85 × 10^8^	
1400	35.0	7.05 × 10^8^	
1500	36.5	7.25 × 10^8^	7.22 × 10^−9^
**Liquid**			
1400			6.89 × 10^−9^
1500	135	7.55 × 10^8^	
1800		8.05 × 10^8^	
2000	135		6.59 × 10^−9^

**Table 5:** Temperature dependent electrical material conductivity $\sigma_{e}$.

Temperature	Electrical Conductivity
°C	1/mm mOhm
20	1.7
200	1.5
400	1.4
800	1.2
1500	1.1
1600	1.1

**Table 6:** Temperature dependent Poisson’s ratio of the low temperature phase and austenite as well as the phase independent Young’s modulus.

Temperature	Low Temperature Phase Poisson’s Ratio	Austenite Poisson’s Ratio	Young’s Modulus
[°C]	ν [-]	ν [-]	[N/mm^2^]
25	0.296	0.294	2.20 × 10^5^
200	0.302	0.305	2.10 × 10^5^
400	0.313	0.317	1.70 × 10^5^
600	0.324	0.329	1.15 × 10^5^
700	0.329	0.335	7.50 × 10^4^
800	0.334	0.341	2.80 × 10^4^
900	0.339	0.346	1.00 × 10^4^
1000	0.344	0.353	3.00 × 10^3^
1200	0.354	0.365	1.67 × 10^3^

## Data Availability

The raw/processed data required to reproduce these findings cannot be shared at this time due to technical or time limitations.
